# State-level economic uncertainty and cardiovascular disease deaths: evidence from the United States

**DOI:** 10.1007/s10654-023-01076-3

**Published:** 2023-11-15

**Authors:** Ilias Kyriopoulos, Sotiris Vandoros, Ichiro Kawachi

**Affiliations:** 1 Department of Health Policy, The London School of Economics and Political Science, Houghton Street, London, WC2A 2AE UK; 2 UCL Global Business School for Health, University College London, London, UK; 3 Harvard T.H. Chan School of Public Health, Harvard University, Cambridge, UK

**Keywords:** Economic uncertainty, Economic conditions, Cardiovascular Disease, United States

## Abstract

The relationship between economic recessions and cardiovascular mortality has been widely explored. However, there is limited evidence on whether economic uncertainty alone is linked to cardiovascular disease deaths. This study examines the association between economic uncertainty and mortality from diseases of the circulatory system in the United States. We obtained monthly state-level mortality data from 2008 to 2017 and used indices capturing economic uncertainty from national/international sources and local sources. Panel data modelling was used to account for unobserved time-invariant differences between the states. Our findings suggest that economic uncertainty is independently linked to cardiovascular mortality. Uncertainty arising from national/international sources is associated with cardiovascular deaths, whereas the respective index capturing uncertainty from state/local sources is not. Deaths respond asymmetrically with respect to uncertainty fluctuations – with high levels of uncertainty driving the association. One- and two-month lagged uncertainty levels are also associated with mortality. Several robustness checks further validate the baseline findings. Overall, economic uncertainty is an independent predictor of cardiovascular mortality which appears to act as a psychosocial stressor and a short-term trigger. Public health strategies for cardiovascular disease need to consider factors driving economic uncertainty. Preventive measures and raising awareness can intensify in periods of economic uncertainty.

**Supplementary Information:**

The online version contains supplementary material available at 10.1007/s10654-023-01076-3.

## Background

Over the last decade, and following the global financial crisis in 2008, rising political polarisation, geopolitical tensions and the COVID-19 pandemic, there are salient concerns about escalating levels of economic uncertainty globally [1, 2]. In this context, the IMF Director recently identified that increasing uncertainty will be the “theme at the outset of the new decade” [3]. Uncertainty can be defined as “peoples’ inability to forecast the likelihood of events happening” [4]. Economic uncertainty is widespread, multifaceted, and substantially different from economic conditions that have already surfaced.

Contrary to actual financial events, such as unemployment or recessions, economic uncertainty echoes ambiguity and concerns about future events that may or may not happen. Uncertainty is not necessarily associated with changes in households’ financial situation or actual financial hardship. Further, although it correlates with the general economic environment, its trends often exhibit substantially different patterns [5].

A strand of the literature has explored how economic conditions may affect cardiovascular disease and deaths. Most studies have found that cardiovascular disease mortality decreases during economic downturns [6, 7], while others provide evidence of countercyclicality of cardiovascular outcomes [8, 9]. The effect of recessions on risk of cardiovascular death is also found to be heterogeneous with respect to employment status [10]. Going beyond the role of economic conditions and events that have already materialised (e.g. unemployment, income loss), evidence on whether and how uncertainty about future economic events alone impacts cardiovascular disease is limited and targeted to the role of job insecurity (which is not the same as job loss). Studies have found a modest association between job insecurity and cardiovascular disease [11, 12].

Examining and understanding the impact of economic uncertainty (rather than actual economic developments such as unemployment or recessions) on health is a topic that has only recently attracted interest. A body of evidence has revealed that economic uncertainty matters for (some) health indicators, mainly focusing on suicide mortality [13–15], road traffic accidents [16, 17] and newborn health [18]. A recent study used time-series data for England and Wales and showed a strong link between economic uncertainty and cardiovascular disease mortality [19]. Other studies have shown that major events that cause uncertainty may also have an effect on people’s mental health and well-being [20–22]. The growing interest in this topic is possibly driven by the substantial increase in economic uncertainty over the past decades [23], as well as by the methodological advances of its measurement. However, economic uncertainty as a determinant of cardiovascular disease has not been widely explored, despite cardiovascular disease being the world’s leading cause of death, associated with substantial and increasing burden of disease and disability [24].

Apart from the role of chronic stressors and health behaviours, exposure to physical and psychological triggers can precipitate cardiovascular disease events and deaths, e.g., via acute atherosclerotic plaque rupture and thrombosis or cardiac arrhythmias [25, 26]. Contrary to other risk factors which gradually contribute to progressive atherosclerosis, triggers are considered as a final-step pathophysiologic mechanism of cardiovascular events [27]. In this spirit, economic uncertainty may contribute to acute psychosocial stress through two channels. First, it directly acts as an emotional trigger [27]. Second, it increases the likelihood of short-term deterioration of health behaviors (e.g., increased intensity of daily smoking) which may, in turn, trigger acute coronary events [28].

Using state-level panel data from the United States, the objective of this study is to examine and elucidate the link between economic uncertainty and cardiovascular mortality. In this context, we explore the role of economic uncertainty as a psychosocial stressor and a potential short-term trigger rather than the impact of actual economic conditions.

This study contributes to existing literature in several ways. First, it studies whether there is an association between economic uncertainty and cardiovascular deaths in the US, rather than that of economic conditions that have already emerged, thus contributing to the existing literature on economic uncertainty and health [13–16, 29]. Second – as opposed to the only relevant study on uncertainty and cardiovascular mortality [19] that employed time-series data at the national level – we rely on sub-national data and exploit variation across states and over time. This allows us to draw on panel data approaches, thus accounting for unobserved state-specific heterogeneity. Third, from a conceptual perspective, this is the first study that provides a better understanding of the sources of economic uncertainty that tend to influence cardiovascular deaths.

## Methods

### Data

We used monthly state-level data for deaths from diseases of the circulatory system (ICD-10 code: I00-I99) for the period 2008–2017, as reported by the Centers for Disease Prevention & Control (CDC). Our analysis therefore focuses on mortality rather than other measures of cardiovascular health [30]. Monthly state unemployment rates were retrieved from the Bureau of Labour Statistics. We also obtained data for GDP growth and poverty rates from the Bureau of Economic Analysis. Population data were obtained from the US Census Bureau. Drawing on a recently published OECD/Eurostat report [31], we also distinguish deaths into avoidable and non-avoidable causes. Avoidable deaths are derived from the OECD/Eurostat list of ICD-10 causes, which are either considered as treatable from health care activities or preventable by specific public health interventions. These only include deaths of under 75-year-olds.

To capture economic uncertainty, we rely on the Economic Policy Uncertainty (EPU) indices measured at a state-month level. The approach for constructing state-specific EPU indices builds on earlier work [1] and draws on the digital archives of almost 3,500 local newspapers published on daily or weekly basis and circulated throughout the states. The indices are constructed based on the number of articles that contain specific economic, uncertainty and policy terms. In particular, they capture the levels of economic uncertainty by tracking and measuring the frequency of articles with terms relating to economic policy changes and uncertainty (e.g. ‘economic’, ‘economy’ and ‘uncertain’, ‘uncertainties’, uncertainty’). EPU indices draw on the seminal studies by Baker et al. (2016) and Baker et al. (2022). As explained in the relevant studies [1, 32], the accuracy and potential bias of the index have been evaluated and extensively validated during its conceptualisation and development. EPU indices have been recently used in economics, finance [2, 33–37], and epidemiology and public health [13–15, 18, 29], and their empirical application has transformed the research field for economic uncertainty [37]. A potential issue with the use of a news-based index relates to potential bias and political slant of the newspapers. Drawing on approaches for measuring newspapers slant, previous studies have tested and empirically scrutinised this scenario, concluding that this is not the case and confirming the validity of the EPU index [1, 32, 38]. The fact that newspapers are used to create the economic index, does not mean that this is the sole channel via which the public becomes aware of economic uncertainty. Some people might not read newspapers, but they may feel uncertain after receiving information via other channels. The uncertainty index thus captures the level of uncertainty, that some people may become aware of through other sources. In addition, a detailed explanation on why newspapers are used to construct the index can be found in the papers published by the creators of the index [1, 32, 39]. While economic uncertainty might originate from a number of economic or non-economic factors, the index captures how any factor translates particularly into economic uncertainty, rather than uncertainty on other dimensions.

Previous studies on the link between economic uncertainty and health have used a single measure of economic uncertainty at the country level. Contrary to these papers, we employ a recently developed measure that varies by state, which also allows us to disentangle state/local from national/international drivers of economic uncertainty [1, 32]. We draw on two state-level EPU indices. The first focuses on economic uncertainty within a state that arises from national sources and events. Its construction further draws on terms of national interest such as national elections, federal departments, agencies, and regulators. The second measures uncertainty levels stemming from state and local sources and builds on terms of state-specific executive, legislative and regulatory bodies. A detailed description of the approach and the terms used to flag relevant articles is presented in Baker et al. (2022).

### Empirical approach

Our analysis is based on a panel data econometric approach, using monthly observations for 51 US states as the level of analysis. Panel data exhibit several advantages over cross-sectional or time series data, which have been extensively analysed in econometric literature [40]. They allow us to control for unobserved time-invariant differences between the states and to eliminate potential bias due to omitted time-invariant state-specific characteristics.

The dependent variable is the number of deaths in each state and month. The two independent variables of interest capture the national and the state sources of economic uncertainty respectively. EPU indices are standardised by dividing them with their respective standard deviation for each state [41]. Last, we control for a vector of independent variables, which includes unemployment, GDP growth, population size, consumer price index and poverty rate. We also controlled for year and month dummies to account for potential seasonality. Robust standard errors, clustered at the state level, are reported throughout.

To examine the association between the different types of EPU and the number of cardiovascular deaths, we rely on a Poisson regression model, given that the number of deaths is a discrete variable taking positive values. Contrary to linear probability models – which rely on assumptions about continuous outcomes that are normally distributed – Poisson models fit the number of occurrences of an event (in this case, deaths). They have been widely used in empirical analysis of count data [42–44], including data on the number of deaths [45–47]. The econometrics literature has extensively discussed the application of Poisson models in empirical research, and has explained aspects such as the interpretation of relevant coefficients [48, 49]. Apart from the estimates for the total population, we also perform additional analyses, providing relevant evidence for cardiovascular deaths by gender. Since our baseline model uses a given month’s uncertainty index and the same month’s mortality, our analysis focuses on the short-term association. Some additional regressions also study whether there is a lagged association, but this is again limited only to a few months. The uncertainty index is reported at the state level, and therefore captures uncertainty for the whole state. As this is a macro-level index, we do not know the level of uncertainty that each individual experiences.

Next, we also test potential non-linear links between economic uncertainty and cardiovascular deaths, by splitting EPU indices into quintiles. In doing so, earlier methodological approaches for estimating non-linear relationships are adopted [15, 41]. Our point of departure is that different levels of economic uncertainty might have differential impact on deaths, demonstrating an asymmetric response of cardiovascular mortality to its risk factors. After splitting EPU indices into quintiles, we control for the first and fifth quintile of the distributions. The remaining quintiles (i.e., the three middle ones) for both EPU indices are omitted and used as reference categories.

Apart from the baseline estimates, we also perform additional analyses to test the sensitivity of our results to different estimation strategies or econometric specifications. First, we employ a Negative Binomial model, which is a suitable alternative to Poisson for modelling count data [43, 50]. Second, we further control for linear and quadratic time trends to capture the potential trajectory of the outcome variable over time. This empirical exercise serves as an additional check, in which we examine the extent to which the direction and statistical significance of the estimates remain unchanged after the inclusion of time trends. Third, we use the logarithmic transformation of the dependent variable and estimate fixed effects and random effects models. Last, we exclude the bottom and top 1% and 5% of the observations (depending on the values of economic uncertainty and those of the outcome variable), to explore the extent to which our estimates are driven or explained by outliers.

## Results

Table 1 shows the baseline regression estimates derived from a Poisson regression model, which is a suitable approach for modelling count data such as the number of cardiovascular deaths. Column 1 presents the estimates after controlling for unemployment, GDP growth, population and the relevant month and year dummies. In Column 2, the model also includes poverty rate and Consumer Price Index (CPI) as independent variables. In Columns 3 and 4 we present the estimates from a Negative Binomial model with the same regressors.

**Table 1:** Baseline regression results: economic uncertainty and cardiovascular deaths

	(1)	(2)	(3)	(5)
	Total	Total	Total	Total
	Poisson	Poisson	Negative Binomial	Negative Binomial
EPU-N	0.0034***	0.0034***	0.0037***	0.0037***
	(0.0010)	(0.0010)	(0.0008)	(0.0008)
EPU-S	0.0005	0.0000	0.0002	-0.0002
	(0.0012)	(0.0012)	(0.0008)	(0.0008)
Main controls	Yes	Yes	Yes	Yes
Additional controls	No	Yes	No	Yes
Month FE	Yes	Yes	Yes	Yes
Year FE	Yes	Yes	Yes	Yes
Observations	6,120	6,120	6,120	6,120
Number of States	51	51	51	51

Note: Main controls include unemployment, GDP growth and population. Additional controls also include CPI and poverty rate. GDP: Gross Domestic Product; CPI: Consumer Price Index: EPU-N: economic policy uncertainty index arising from national/international sources; EPU-S: economic policy uncertainty index arising from state/local sources; FE: fixed effects. Regression coefficients and standard errors (in parentheses) are reported

*p < 0.10, **p < 0.05, ***p < 0.01

Our findings reveal that national and international sources of economic uncertainty are positively associated with cardiovascular disease deaths (Coef.: 0.0034; p < 0.01). This is not the case for the economic uncertainty induced by state and local sources, the coefficient of which is not statistically significant at conventional levels of significance (Coeff.: 0.0005; p > 0.10). These findings remain insensitive (both in terms of magnitude and significance) after the inclusion of additional controls, such as the consumer price index and poverty rate. Our baseline specification demonstrates that a one standard deviation increase in economic uncertainty index driven from national/international sources is associated with an increase in the number of deaths by 0.34%.

In Table 2, we present the estimates derived from our baseline specification, using the number of deaths among females (Columns 1–2) and males respectively (Columns 3–4). In Column 1, the model includes the main set of controls (i.e., unemployment, GDP growth, population), whereas the model presented in Column 2 also controls for poverty rate and CPI. Columns 3 and 4 show the estimates for males, following the same approaches with Columns 1 and 2 respectively. As revealed in Table 2, our estimates do not provide evidence of heterogeneity with respect to gender. The link between economic uncertainty and cardiovascular disease deaths holds for both females and males, with the coefficient of interest being only marginally greater for females. Similar to the results for the total deaths, only national/international sources of economic uncertainty are associated with cardiovascular mortality in males and females.

**Table 2:** Regression results by gender: economic uncertainty and cardiovascular deaths

	(1)	(2)	(3)	(4)
	Female	Female	Male	Male
	Poisson	Poisson	Poisson	Poisson
EPU-N	0.0038***	0.0037***	0.0031***	0.0030***
	(0.0011)	(0.0011)	(0.0011)	(0.0011)
EPU-S	0.0012	0.0007	-0.0003	-0.0007
	(0.0012)	(0.0012)	(0.0014)	(0.0014)
Main controls	Yes	Yes	Yes	Yes
Additional controls	No	Yes	No	Yes
Month FE	Yes	Yes	Yes	Yes
Year FE	Yes	Yes	Yes	Yes
Observations	6,120	6,120	6,120	6,120
Number of States	51	51	51	51

Note: Main controls include unemployment, GDP growth and population. Additional controls also include CPI and poverty rate. GDP: Gross Domestic Product; CPI: Consumer Price Index: EPU-N: economic policy uncertainty index arising from national/international sources; EPU-S: economic policy uncertainty index arising from state/local sources; FE: fixed effects. Regression coefficients and standard errors (in parentheses) are reported

*p < 0.10, **p < 0.05, ***p < 0.01

Next, we examine whether cardiovascular disease mortality responds asymmetrically with respect to changes in economic uncertainty. Table 3 shows that the dummy variable capturing the top quintile of national economic uncertainty is strongly associated with cardiovascular disease deaths. High levels of economic uncertainty therefore matter for both males and females. These estimates further confirm our baseline findings for the sources of economic uncertainty. Our findings suggest that higher levels of economic uncertainty (i.e., in fifth quintile) are associated with a 0.6% increase in cardiovascular deaths. These findings are further confirmed by the analysis presented in eFigure 1 in the Online Supplementary Material, which shows significant difference in cardiovascular mortality rates between the bottom and top quintile of economic uncertainty.

**Table 3:** Regression results: non-linear relationship between economic uncertainty and cardiovascular deaths

	(1)	(2)	(3)
	Total	Female	Male
	Poisson	Poisson	Poisson
EPU-N: Bottom quintile	-0.0028*	-0.0025	-0.0030
	(0.0017)	(0.0019)	(0.0021)
EPU-N: Top quintile	0.0060***	0.0083***	0.0035**
	(0.0016)	(0.0018)	(0.0018)
EPU-S: Bottom quintile	-0.0026	-0.0028	-0.0023
	(0.0023)	(0.0027)	(0.0023)
EPU-S: Top quintile	0.0001	0.0010	-0.0010
	(0.0018)	(0.0020)	(0.0019)
Controls	Yes	Yes	Yes
Month FE	Yes	Yes	Yes
Year FE	Yes	Yes	Yes
Observations	6,120	6,120	6,120
Number of States	51	51	51

Note: Controls include unemployment, GDP growth, population, CPI, and poverty rates. GDP: Gross Domestic Product; CPI: Consumer Price Index: EPU-N: economic policy uncertainty index arising from national/international sources; EPU-S: economic policy uncertainty index arising from state/local sources; FE: fixed effects. Regression coefficients and standard errors (in parentheses) are reported

*p < 0.10, **p < 0.05, ***p < 0.01

Individuals might not always be directly affected by specific events or conditions. It might take some time to feel and process the implications of economic uncertainty, which might therefore have a lasting impact. We capture this by exploring potential lags between economic uncertainty and the outcome of interest. The estimates presented in Table 4 suggest that one-month and two-month lag of uncertainty matter for cardiovascular disease deaths. In particular, the coefficients of the lagged economic uncertainty from national sources (see Columns 1–2 in Table 4) are positive and statistically significant. This finding demonstrates that it is not only the contemporaneous economic uncertainty that matters for cardiovascular deaths, but also that of the previous period. Similar to the findings reported above, it is only economic uncertainty from national/international sources that is linked with cardiovascular mortality, and not the one arising from state/local sources. In particular, a one standard deviation increase in lagged economic uncertainty in economic uncertainty index is associated with an increase in the number deaths by almost 0.3%. We also find evidence of similar lagging relationship when focusing on female and male deaths (see eTable 2). Apart from this analysis, we also included all five lags as covariates in one model (eTable 3).

**Table 4:** Regression results: lagging relationship between economic uncertainty and cardiovascular deaths

	(1)	(2)	(3)	(4)	(5)
	Total	Total	Total	Total	Total
	Poisson	Poisson	Poisson	Poisson	Poisson
	1-month	2-month	3-month	4-month	5-month
Lagged EPU-N	0.0036***	0.0027**	0.0017	-0.0018	-0.0010
	(0.0012)	(0.0011)	(0.0011)	(0.0013)	(0.0012)
Lagged EPU-S	-0.0003	0.0019	0.0011	0.0021*	0.0018
	(0.0013)	(0.0014)	(0.0011)	(0.0011)	(0.0012)
Controls	Yes	Yes	Yes	Yes	Yes
Month FE	Yes	Yes	Yes	Yes	Yes
Year FE	Yes	Yes	Yes	Yes	Yes
Observations	6,069	6,018	5,967	5,916	5,865
Number of States	51	51	51	51	51

Note: Controls include unemployment, GDP growth, population, CPI, and poverty rates. GDP: Gross Domestic Product; CPI: Consumer Price Index: EPU-N: economic policy uncertainty index arising from national/international sources; EPU-S: economic policy uncertainty index arising from state/local sources; FE: fixed effects. Regression coefficients and standard errors (in parentheses) are reported

*p < 0.10, **p < 0.05, ***p < 0.01

In addition to the models presented above, we also follow different approaches to check the robustness of the baseline findings. First, although the Poisson model is our baseline specification, we perform an additional analysis using a Negative Binomial model, which confirms our main results (see Columns 3–4 in Table 1). Second, the baseline specification is modified by including a time trend and, subsequently, an additional quadratic time trend. The estimates provide strong evidence of a link between national/international sources of economic uncertainty and cardiovascular disease mortality (see eTable 4). In addition, the size of the coefficients are similar to the one derived from our baseline models. Third, we use the logarithmic transformation of deaths as dependent variable and employ fixed effects and random effects models. The results, presented in eTable 5 in the Supplementary Material, further confirm our baseline findings. Fourth, we change the transformation method of the independent variable of interest. As a standardisation method for our baseline specification, EPU indices were divided by the standard deviation. In addition to this approach, alternative models are estimated, in which a logarithmic transformation is applied to the values of EPU indices. According to the estimates presented in eTable 6 in the Supplementary Material, the results are consistent. Fifth, we conduct an internal check of the validity of the findings by modelling other causes of mortality, which are unlikely to be linked with uncertainty. We perform a placebo test on outcomes for which we would not expect an association with economic uncertainty (i.e. deaths from cancer or diseases of the musculoskeletal system). Indeed, we find that these mortality causes are not influenced by economic uncertainty (eTable 7 in the Supplement). Sixth, we examine the association between economic uncertainty and avoidable and non-avoidable cardiovascular mortality, as defined by the relevant OECD/Eurostat list of avoidable causes of death. As noted above, this list comprises of specific causes of mortality, and focuses only on deaths of those aged less than 75 years. According to the estimates presented in eTable 8, economic uncertainty from national sources is correlated with the number of non-avoidable deaths. Last, we trim the observations whose values of economic uncertainty are in the bottom and top 1% and 5%. The results, presented in eTable 9 (Columns 1–2), confirm our baseline estimates. Our findings also remain consistent after excluding the observations with low and high number of cardiovascular deaths (see Columns 3–4 in eTable 9). By performing this analysis, we rule out the possibility that our baseline findings are driven by outliers.

## Discussion

Using state-level data from the United States, this study shows that economic uncertainty is independently associated with deaths from cardiovascular diseases and is detrimental for both females and males. It reveals yet another cardiovascular risk factor, and highlights the importance of uncertainty in addition to actual economic developments and conditions. It thus demonstrates the role of uncertainty as a stressor and trigger for cardiovascular mortality. The results add to existing studies on the role of economic uncertainty when it comes to health outcomes, such as suicides, newborn health, and motor vehicle collisions [13–16, 18].

When distinguishing the different sources of economic uncertainty, we demonstrate that uncertainty indices capturing national and international sources seem to matter the most – as opposed to more local sources of uncertainty. This finding is consistent across different modelling approaches. There are two possible interpretations for this. First, this might have to do with the absolute magnitude of national-level uncertainty, where events affecting the whole country or that even have global effects, are likely to be more important and may have more serious economic consequences. Second, events at the national or international level might be considered as beyond the remit of the local authorities at the state level, and thus less under control. State inhabitants might thus feel less able to influence or even understand the source and type of economic uncertainty from national/international sources, or the likely financial outcomes – thus adding to their perception of insecurity. This can be partly explained by the concept of proximity to power and political accessibility, especially given that people in the US tend to trust the local and state governments more than the federal one [51]. In this light, economic policy uncertainty may increase stress and trigger cardiovascular events to a larger extent in cases where people are not confident with or do not trust those in charge of handling the relevant policy issues. By contrast, people may cope with increased levels of uncertainty more effectively when it stems from sources of political power they trust more (i.e., state, and local sources). Economic uncertainty from national sources (as compared to that from more localized sources) may lead to more diffused levels of fear, stress and anxiety, and therefore lower appraisals of controllability. This difference between state-level and national sources of uncertainty adds to previous findings that focus on the impact of large-scale events of national interest that trigger uncertainty. For example, evidence suggests that several health indicators have deteriorated immediately after national-level events that induce uncertainty to a large population share, such as the 2016 presidential elections in the US [52, 53], the Brexit referendum [20, 21], or terrorist attacks [22, 54].

Deaths appear to respond asymmetrically with respect to uncertainty fluctuations – with high levels of uncertainty driving the association. In other words, extreme levels of economic uncertainty influence cardiovascular disease deaths the most. In a similar spirit, another study examined the impact of stock market changes on hospital admissions, and showed that only large market declines matter [41]. Suicide rates have also been found to respond asymmetrically to changes in economic uncertainty [15]. This finding can be partly interpreted considering individuals’ tendency to give more weight and focus on negative rather than positive news [55]. This presence of negativity bias essentially demonstrates that the negative influence of economic uncertainty on cardiovascular is not necessarily compensated by periods of limited uncertainty.

We further provide evidence on the dynamics of this link, showing that two-month, one-month lagged and contemporaneous uncertainty levels are associated with cardiovascular deaths, while there is little or no association beyond that time horizon.

As cardiovascular disease is a leading cause of death, preventive measures can play an important role in reducing mortality. Counselling services, campaigns raising awareness on the symptoms of heart attacks or strokes as well as information on how to respond, can intensify in periods of national economic uncertainty. Our findings also suggest that careful political communication of economic policy aspects also matters, as it is often the real cause behind escalating levels of economic uncertainty. In essence, the way through which political and economic messages are conveyed is important for cardiovascular disease and mortality and public health in general. Overall, this study has highlighted yet another risk factor, and opens another path to understand the relationship between economic conditions and cardiovascular mortality.

This study is not without limitations. First, the present analysis draws on aggregate data, which does not allow us to control for individual-level risk factors, such as hypertension, comorbidities, lifestyle, and obesity, among others. Second, it only focuses on deaths, excluding non-fatal cardiovascular events due to data availability constraints. Examining how economic uncertainty influences morbidity would allow us to provide a more comprehensive overview of the effects on cardiovascular disease in general. Third, we cannot identify potential heterogeneity with respect to socioeconomic characteristics, although it is likely that more vulnerable groups are disproportionately affected. Overall, using the EPU index has several advantages. It captures uncertainty, i.e. it relates to events that have not occurred, as opposed to economic growth or unemployment, which capture actual economic conditions. In other words, the uncertainty index echoes ambiguity and concerns about future events that may or may not happen, and is not necessarily associated with current changes in households’ financial situation or actual financial hardship. Further, it is available at state level and monthly frequency. This measure is also continuous, which makes it easier to use empirically and provides more information than ordinal outcomes. However, as with any economic indicator, it is available at the aggregate level, so we cannot perform a micro-level analysis.

Cardiovascular disease is the leading cause of mortality globally. Understanding what drives cardiovascular deaths is particularly important and our study reveals the role of uncertainty in cardiovascular deaths.

### Electronic supplementary material

Below is the link to the electronic supplementary material.

Supplementary Material 1

## Data Availability

The data used are publicly available.
