# Supplementary material for: State-level economic uncertainty and cardiovascular disease deaths: evidence from the United States
Source: Eur J Epidemiol. 2023 Nov 15;38(11):1175–83. doi: 10.1007/s10654-023-01076-3 (PMC10663230; doi:10.1007/s10654-023-01076-3)
Supplement: Supplementary file 1 — Supplementary Material 1 [file 10654_2023_1076_MOESM1_ESM.pdf]

State-level economic uncertainty and cardiovascular deaths: evidence from the United States

Online Supplement

eTable 1: Summary statistics

Variable	Observations	Mean	Standard Deviation
**Total deaths**	6,120	1322.5	1396.6
**Female deaths**	6,120	662.0	700.7
**Male deaths**	6,120	660.5	698.3
**EPU-N**	6,120	106.0	65.7
**EPU-S**	6,120	75.8	53.5
**Unemployment**	6,120	6.5	2.2
**GDP growth**	6,120	1.4	5.2
**Population (million)**	6,120	6.2	6.9
**CPI**	6,120	229.4	10.4
**Poverty rate**	6,120	13.5	3.4

eTable 2: Lags of economic uncertainty and cardiovascular deaths, by gender

Panel A: Females

	(1) Female	(2) Female	(3) Female	(4) Female	(5) Female
	Poisson	Poisson	Poisson	Poisson	Poisson
	1-month	2-month	3-month	4-month	5-month
Lagged EPU-N	0.0041***	0.0029**	0.0016	-0.0021	-0.0009
	(0.0014)	(0.0012)	(0.0012)	(0.0015)	(0.0012)
Lagged EPU-S	-0.0005	0.0021	0.0013	0.0025**	0.0017
	(0.0015)	(0.0015)	(0.0012)	(0.0012)	(0.0013)
Controls	Yes	Yes	Yes	Yes	Yes
Month FE	Yes	Yes	Yes	Yes	Yes
Year FE	Yes	Yes	Yes	Yes	Yes
Observations	6,069	6,018	5,967	5,916	5,865
Number of States	51	51	51	51	51

Panel A: Males

	(1) Male	(2) Male	(3) Male	(4) Male	(5) Male
	Poisson	Poisson	Poisson	Poisson	Poisson
	1-month	2-month	3-month	4-month	5-month
Lagged EPU-N	0.0030**	0.0024**	0.0018	-0.0016	-0.0011
	(0.0012)	(0.0012)	(0.0011)	(0.0013)	(0.0014)
Lagged EPU-S	-0.0002	0.0016	0.0010	0.0016	0.0019
	(0.0014)	(0.0016)	(0.0013)	(0.0013)	(0.0012)
Controls	Yes	Yes	Yes	Yes	Yes
Month FE	Yes	Yes	Yes	Yes	Yes
Year FE	Yes	Yes	Yes	Yes	Yes
Observations	6,069	6,018	5,967	5,916	5,865
Number of States	51	51	51	51	51

Note: Controls include unemployment, GDP growth and population, CPI, and poverty rate. EPU-N: economic policy uncertainty index arising from national/international sources; EPU-S: economic policy uncertainty index arising from state/local sources; FE: fixed effects. Regression coefficients and standard errors (in parentheses) are reported.

*p<0.10, **p<0.05, ***p<0.01.

eTable 3: Lags of economic uncertainty and cardiovascular deaths, additional analysis

	(1) Total	(2) Female	(3) Male
	Poisson	Poisson	Poisson
EPU-N (Lag-1 month)	0.0031***	0.0039***	0.0023**
	(0.0009)	(0.0011)	(0.0010)
EPU-N (Lag-2 month)	0.0013	0.0014	0.0012
	(0.0009)	(0.0010)	(0.0011)
EPU-N (Lag-3 month)	0.0012*	0.0008	0.0016*
	(0.0007)	(0.0010)	(0.0009)
EPU-N (Lag-4 month)	-0.0033***	-0.0037***	-0.0029***
	(0.0010)	(0.0013)	(0.0011)
EPU-N (Lag-5 month)	-0.0009	-0.0008	-0.0010
	(0.0010)	(0.0010)	(0.0012)
EPU-S (Lag-1 month)	-0.0007	-0.0009	-0.0005
	(0.0008)	(0.0011)	(0.0010)
EPU-S (Lag-2 month)	0.0016*	0.0020*	0.0012
	(0.0010)	(0.0012)	(0.0011)
EPU-S (Lag-3 month)	0.0003	0.0005	0.0001
	(0.0009)	(0.0010)	(0.0010)
EPU-S (Lag-4 month)	0.0015*	0.0020*	0.0010
	(0.0009)	(0.0010)	(0.0010)
EPU-S (Lag-5 month)	0.0016	0.0014	0.0018
Controls	Yes	Yes	Yes
Month FE	Yes	Yes	Yes
Year FE	Yes	Yes	Yes
Observations	5,865	5,865	5,865
Number of StateCode	51	51	51

Note: Controls include unemployment, GDP growth and population, CPI, and poverty rate. GDP: Gross Domestic Product; CPI: Consumer Price Index: EPU-N: economic policy uncertainty index arising from national/international sources; EPU-S: economic policy uncertainty index arising from state/local source; FE: fixed effects. Regression coefficients and standard errors (in parentheses) are reported.

*p<0.10, **p<0.05, ***p<0.01.

eTable 4: Economic uncertainty and cardiovascular deaths (controlling for time trends)

	(1)<br>Total	(2)<br>Total	(3)<br>Total	(4)<br>Total
	Poisson	Poisson	Poisson	Poisson
EPU-N	0.0046***	0.0037***	0.0052***	0.0036***
	(0.0017)	(0.0011)	(0.0018)	(0.0011)
EPU-S	-0.0015	0.0006	-0.0016	-0.0002
	(0.0022)	(0.0012)	(0.0022)	(0.0013)
Time trend	0.0019***	0.0011***	-0.0251***	-0.0104***
	(0.0005)	(0.0002)	(0.0076)	(0.0025)
Quadratic time trend			0.0000***	0.0000***
			(0.0000)	(0.0000)
Controls	Yes	Yes	Yes	Yes
Month FE	No	Yes	No	Yes
Year FE	Yes	No	Yes	No
Observations	6,120	6,120	6,120	6,120
Number of StateCode	51	51	51	51

Note: Controls include unemployment, GDP growth and population, CPI, and poverty rate. GDP: Gross Domestic Product; CPI: Consumer Price Index: EPU-N: economic policy uncertainty index arising from national/international sources; EPU-S: economic policy uncertainty index arising from state/local sources; FE: fixed effects. Regression coefficients and standard errors (in parentheses) are reported.

*p<0.10, **p<0.05, ***p<0.01.

eTable 5: Economic uncertainty and cardiovascular deaths (fixed and random effects models)

	(1)<br>Total	(2)<br>Total	(3)<br>Total	(4)<br>Total
	Fixed Effects	Fixed Effects	Random Effects	Random Effects
EPU-N	0.0040***	0.0039***	0.0040***	0.0039***
	(0.0012)	(0.0012)	(0.0012)	(0.0012)
EPU-S	0.0003	-0.0001	0.0004	0.0000
	(0.0014)	(0.0015)	(0.0014)	(0.0015)
Main controls	Yes	Yes	Yes	Yes
Additional controls	No	Yes	No	Yes
Month FE	Yes	Yes	Yes	Yes
Year FE	Yes	Yes	Yes	Yes
Observations	6,120	6,120	6,120	6,120
R-squared	0.5867	0.5875		
Number of States	51	51	51	51

Note: Main controls include unemployment, GDP growth and population. Additional controls also include CPI and poverty rate. GDP: Gross Domestic Product; CPI: Consumer Price Index: EPU-N: economic policy uncertainty index arising from national/international sources; EPU-S: economic policy uncertainty index arising from state/local sources; FE: fixed effects. Regression coefficients and standard errors (in parentheses) are reported.

*p<0.10, **p<0.05, ***p<0.01.

eTable 6: Economic uncertainty and cardiovascular deaths (other specifications)

	(1)<br>Total	(2)<br>Total	(4)<br>Total	(5)<br>Total
	Poisson	Negative Binomial	Fixed Effects	Random Effects
Log EPU-N	0.0044**	0.0048***	0.0049***	0.0049***
	(0.0021)	(0.0012)	(0.0014)	(0.0014)
Log EPU-S	0.0011	0.0003	-0.0001	-0.0000
	(0.0022)	(0.0012)	(0.0022)	(0.0022)
Controls	Yes	Yes	Yes	Yes
Month FE	Yes	Yes	Yes	Yes
Year FE	Yes	Yes	Yes	Yes
Observations	6,120	6,120	6,120	6,120
Number of States	51	51	51	51
R-squared			0.5872	

Note: Controls include unemployment, GDP growth and population, CPI, and poverty rate. In Model 4, we have controlled for the natural logarithm of EPU indices, instead of standardising by the standard deviation. GDP: Gross Domestic Product; CPI: Consumer Price Index: EPU-N: economic policy uncertainty index arising from national/international sources; EPU-S: economic policy uncertainty index arising from state/local source; FE: fixed effects. Regression coefficients and standard errors (in parentheses) are reported.

*p<0.10, **p<0.05, ***p<0.01.

eTable 7: Economic uncertainty and deaths (placebo outcomes)

	(1)	(2)
	C00-D48	M00-M99
	Poisson	Poisson
EPU-N	0.0004	0.0007
	(0.0008)	(0.0048)
EPU-S	0.0002	-0.0018
	(0.0007)	(0.0138)
Controls	Yes	Yes
Month FE	Yes	Yes
Year YE	Yes	Yes
Observations	6,120	6,120
Number of States	51	51

Note: Controls include unemployment, GDP growth and population, CPI, and poverty rate. GDP: Gross Domestic Product; CPI: Consumer Price Index: EPU-N: economic policy uncertainty index arising from national/international sources; EPU-S: economic policy uncertainty index arising from state/local sources; FE: fixed effects. Regression coefficients and standard errors (in parentheses) are reported.

*p<0.10, **p<0.05, ***p<0.01.

eTable 8: Economic uncertainty, avoidable and non-avoidable deaths

	(1)<br>Non-avoidable causes	(2)<br>Avoidable causes
	Poisson	Poisson
EPU-N	0.0043***	0.0010
	(0.0011)	(0.0016)
EPU-S	-0.0004	0.0009
	(0.0012)	(0.0017)
Controls	Yes	Yes
Month FE	Yes	Yes
Year YE	Yes	Yes
Observations	6,120	6,120
Number of States	51	51

Note: Controls include unemployment, GDP growth and population, CPI, and poverty rate. GDP: Gross Domestic Product; CPI: Consumer Price Index: EPU-N: economic policy uncertainty index arising from national/international sources; EPU-S: economic policy uncertainty index arising from state/local sources; FE: fixed effects. Regression coefficients and standard errors (in parentheses) are reported.

*p<0.10, **p<0.05, ***p<0.01.

eTable 9: Economic uncertainty, avoidable and non-avoidable deaths

	(1) Excl. 1% wrt to EPU-N	(2) Excl. 5% wrt to EPU-N	(3) Excl. 1% wrt to deaths	(4) Excl. 5% wrt to deaths
	Poisson	Poisson	Poisson	Poisson
EPU-N	0.0036***	0.0043***	0.0031***	0.0026**
	(0.0011)	(0.0012)	(0.0010)	(0.0011)
EPU-S	0.0001	0.0000	0.0003	-0.0006
	(0.0013)	(0.0012)	(0.0013)	(0.0010)
Controls	Yes	Yes	Yes	Yes
Month FE	Yes	Yes	Yes	Yes
Year FE	Yes	Yes	Yes	Yes
Observations	5,997	5,508	6,005	5,516
Number of States	51	51	51	49

Note: Controls include unemployment, GDP growth and population, CPI, and poverty rate. GDP: Gross Domestic Product; CPI: Consumer Price Index: EPU-N: economic policy uncertainty index arising from national/international sources; EPU-S: economic policy uncertainty index arising from state/local sources; FE: fixed effects. Regression coefficients and standard errors (in parentheses) are reported. In Column 1 we trim the bottom and top 1% of observations with the lowest and highest values of economic uncertainty. In Column 2, we repeat the same exercise, excluding the bottom and top 5%. In Column 3 (4), we trim the bottom and top 1% (5%) of observations with the lowest and highest values of cardiovascular disease deaths.

*p<0.10, **p<0.05, ***p<0.01.

eFigure 1: Differences in mortality rates across different EPU quintiles

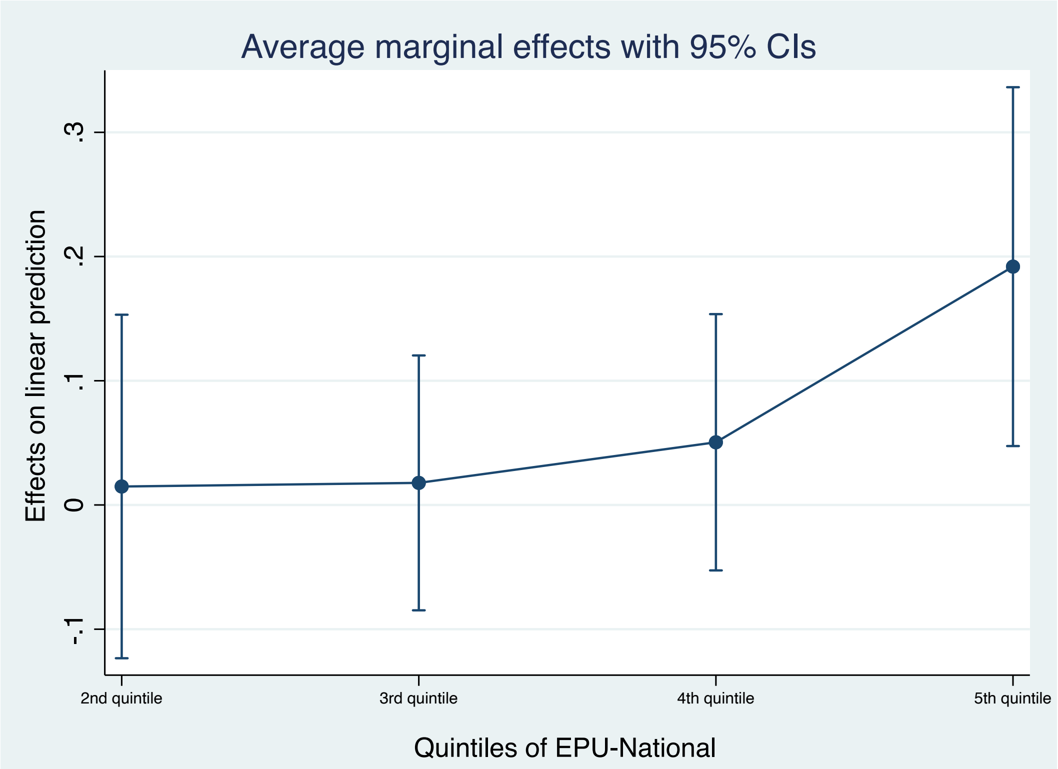

Notes: Estimates of the difference in mortality rate between each quintile of economic uncertainty relative to the first quintile (lower levels of economic uncertainty).
